# 3D neuronal mitochondrial morphology in axons, dendrites, and somata of the aging mouse hippocampus

**DOI:** 10.1016/j.celrep.2021.109509

**Published:** 2021-08-10

**Authors:** Julie Faitg, Clay Lacefield, Tracey Davey, Kathryn White, Ross Laws, Stylianos Kosmidis, Amy K. Reeve, Eric R. Kandel, Amy E. Vincent, Martin Picard

**Affiliations:** 1 Wellcome Centre for Mitochondrial Research, Translational and Clinical Research Institute, Faculty of Medical Sciences, Newcastle University, Newcastle upon Tyne, UK; 2 Electron Microscopy Research Services, Newcastle University, Newcastle, UK; 3 New York State Psychiatric Institute, New York, NY 10032, USA; 4 Zuckerman Mind Brain Behavior Institute, Kavli Institute for Brain Science, Department of Neuroscience, Howard Hughes Medical Institute, Columbia University, New York, NY, USA; 5 Division of Behavioral Medicine, Department of Psychiatry, Columbia University Irving Medical Center, New York, NY, USA; 6 Department of Neurology, The Merritt Center and Columbia Translational Neuroscience Initiative, Columbia University Irving Medical Center, New York, NY, USA; 7 Lead contact

## Abstract

The brain’s ability to process complex information relies on the constant supply of energy through aerobic respiration by mitochondria. Neurons contain three anatomically distinct compartments—the soma, dendrites, and projecting axons—which have different energetic and biochemical requirements, as well as different mitochondrial morphologies in cultured systems. In this study, we apply quantitative three-dimensional electron microscopy to map mitochondrial network morphology and complexity in the mouse brain. We examine somatic, dendritic, and axonal mitochondria in the dentate gyrus and cornu ammonis 1 (CA1) of the mouse hippocampus, two subregions with distinct principal cell types and functions. We also establish compartment-specific differences in mitochondrial morphology across these cell types between young and old mice, highlighting differences in age-related morphological recalibrations. Overall, these data define the nature of the neuronal mitochondrial network in the mouse hippocampus, providing a foundation to examine the role of mitochondrial morpho-function in the aging brain.

## In brief

Faitg et al. use 3D electron microscopy to quantitatively define mitochondrial morphology in the mouse hippocampus with age. Their analysis shows that axonal, dendritic, and somatic mitochondrial morphologies differ between hippocampal CA1 and DG regions and that age is associated with compartment-specific recalibrations in mitochondrial morphology.

## Graphical abstract

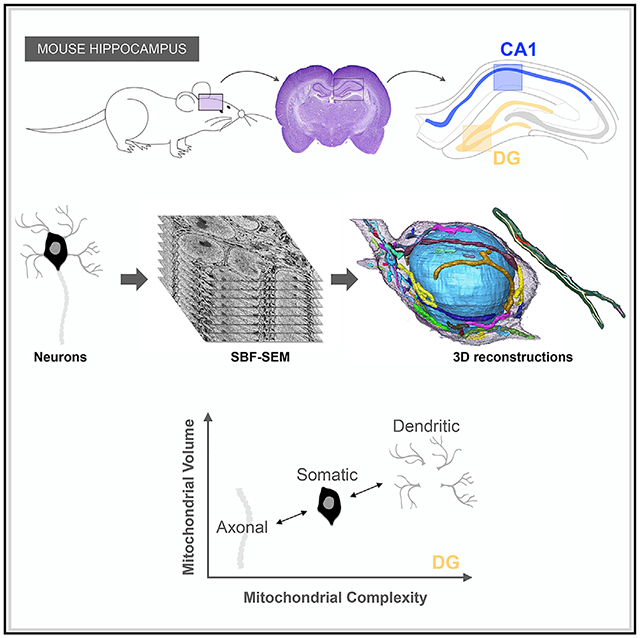

## INTRODUCTION

The mammalian hippocampus is a specialized brain structure underlying the cerebral neocortex with a role in encoding episodic memories and spatial maps, crucial features for organizing adaptive behavior (Hartley et al., 2013). Among the four main subregions of the hippocampus, the cornu ammonis 1 (CA1) and the dentate gyrus (DG) play distinct roles in the conversion and storage of long-term memory (Kandel, 2009; Gilbert et al., 2001), and they are highly specialized in cellular features. For example, the DG contains many small non-pyramidal excitatory neurons, called granule cells, that are much more sparsely active than the large pyramidal neurons of the CA1 (Diamantaki et al., 2016). However, a common property of both DG and CA1 is their high energy requirement.

Brain energy production, among other functions, relies heavily on oxidative phosphorylation within mitochondria, which is necessary for neurotransmitter release and for maintaining neuronal electrochemical gradients (Magistretti and Allaman, 2015). Beyond energy production, mitochondria also perform other key functions that are important for neuronal activity (Youle and van der Bliek, 2012), including steroid hormone synthesis (Selvaraj et al., 2018). In neurons, mitochondria play a critical role in Ca^2+^ buffering (Lewis et al., 2018; Lin et al., 2019), which regulates synaptic transmission and plasticity. Prior to the establishment of terminally differentiated neural structures, the function and positioning of mitochondria also determine axonal and dendritic development (Courchet et al., 2013) and can influence axonal regeneration (Kann and Kovács, 2007; Schon and Przedborski, 2011), illustrating the broad range of effects mitochondria have on neuronal behavior (Lee et al., 2018). Given these diverse roles, it is likely that mitochondria differ between principal cell types in DG and CA1, and they may underlie the relative susceptibility of these areas to age-related changes.

Depending on the tissue, cell type, and location within the cell, mitochondria can vary in morphology from simple spheres to elongated and highly branched organelles (Fawcett, 1981; Bleck et al., 2018; Faitg et al., 2019; Vincent et al., 2019). These morphological transitions take place within seconds to minutes and directly impact mitochondrial functions (Picard et al., 2013). In fact, in humans, disease-causing primary mitochondrial DNA mutations can cause changes in skeletal muscle mitochondrial ultrastructure (Vincent et al., 2016) and organelle morphology (Vincent et al., 2019). This highlights the importance of defining the morphological characteristics of mitochondria in anatomically and functionally distinct cell types, brain regions (DG versus CA1), and subcellular compartments in the mouse.

Differences in neuronal mitochondrial morphology have previously been observed between axonal and dendritic subcompartments. In live imaging of cultured neurons, axonal mitochondria are punctate; in contrast, dendritic mitochondria are more tubular (Lewis et al., 2018). Studies using serial section electron microscopy on fixed brains confirmed the same observation in the primary visual cortex of young mice (Turner et al., 2020), ground squirrels, and young rat hippocampus (Popov et al., 2005). However, we lack a quantitative characterization of the differences in both mitochondrial size and morphology between subcompartments of functionally defined brain areas such as DG and CA1.

Aging may also influence mitochondrial morphology. Compared to young animals, older animals develop mitochondrial ultrastructural alterations and possibly a decrease in mitochondrial function in the brain, including mitochondrial respiratory control ratio (Yao et al., 2010) and antioxidant defenses (Mandal et al., 2012). Morphologically, different studies have reported that with aging, mitochondria are enlarged in the frontal cortex (Navarro and Boveris, 2004) but smaller in the CA1 (Thomsen et al., 2018). Moreover, the effects of aging and disease on neuronal function within different subregions of the hippocampus differ substantially (Barnes, 1994). For example, the CA1 region is one of the regions most affected by Alzheimer’s disease (Braak et al., 2006), whereas with aging the DG exhibits a decline in the neurogenesis of granule cells (Bondareff and Geinisman, 1976; Lemaire et al., 2000). However, no study has previously examined the effect of aging on mitochondrial morphology in both the DG and CA1 of the same animals.

Therefore, we used 3D serial block face scanning electron microscopy (SBF-SEM) (Denk and Horstmann, 2004) to quantitatively image large tissue areas and define mitochondrial morphology in the mouse hippocampus. DG and CA1 mitochondria from dendrites and somata, as well as incoming myelinated axons, were meticulously traced, reconstructed, and quantitatively analyzed in both young and old animals. The strict lamination of these hippocampal areas represents a useful model to study compartmental differences, as it consists of distinct layers containing primarily dendrites, soma, or axons from a relatively homogeneous population of excitatory principal neurons.

## RESULTS

### Compartment-specific imaging of neuronal mitochondria in CA1 and DG regions

**Figure 1.**
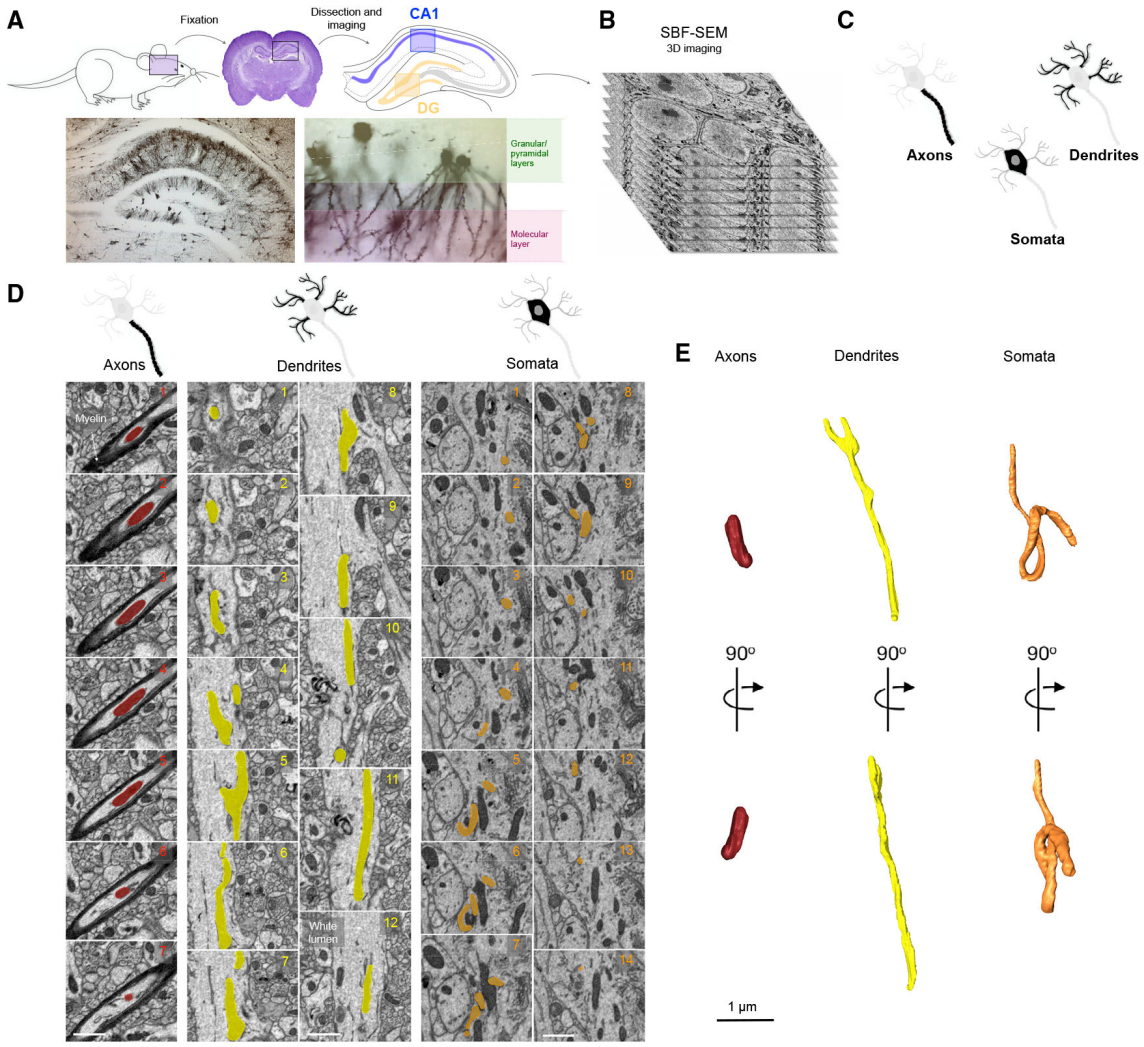
Study design for 3D reconstructions of mitochondria in neuronal subcompartments of the mouse hippocampal DG and CA1 (A) Schematic of study design involving rapid transcardial perfusion and fixation, dissection, coronal sectioning, and imaging of both granular/pyramidal and molecular layers in the DG and CA1. See Figure S1 for details of sub-hippocampal regions of interest selected for imaging. (B) A z stack at electron microscopy (EM) resolution from serial block face scanning EM (SBF-SEM) used for 3D reconstructions. Each image (serial section) is separated by 50 nm, and the total volume includes 400 images. Dimensions are 40 × 40 μm (x/y) and 20 μm (z). (C) Schematic illustrating the three neuronal subcellular compartments analyzed, including axons, dendrites, and somata. (D and E) SBF-SEM images from the CA1 numbered sequentially, showing a highlighted mitochondrion within each neuronal compartment: axons (the dark myelin sheaths), dendrites, and somata (D). (E) 3D reconstruction of mitochondria in (D). The same mitochondria are shown in two different orientations (90° rotation). Scale bars, 1 μm.

Young (4 months, n = 3) and old (18 months, n = 4) C57BL/6J mice were transcardially perfused with fixative after brief anesthesia to preserve *in vivo* mitochondrial morphology. The dorsal hippocampus (bregma = 1.64 mm) was then dissected, embedded, and mounted for SBF-SEM imaging of the DG and CA1 from the same brain section (Figures 1A and 1B). Figure S1 illustrates detailed positioning of the regions of interest (ROIs) imaged to obtain myelinated axons and dendrites (DG or CA1 molecular layers) and somata (DG granular layer, CA1 pyramidal cell layer). For each animal, all mitochondria, the cell membrane, and the nuclear envelope in the soma from three axons, three dendrites, and two to three somata in each hippocampal subfield were fully reconstructed in 3D with an x,y resolution of 7 nm and z resolution of 50 nm (400 serial images per ROI, Figure 1C). The dendrites from the middle of the molecular layer belong to the same cell type as the somata; in contrast, the axons identified in the DG most likely arise and project from the medial entorhinal cortex, whereas CA1 axons likely arise from the CA3, rather than from the principal cell type in the regions themselves. Below we refer to axons, dendrites, and somata by their site of imaging (CA1 or DG), but the origin of axons segments is important to consider when interpreting axonal differences between the areas.

Within the molecular layer, axons were identified by the presence of surrounding myelin while dendrites were illustrated by their non-myelinated shafts with identifiable dendritic spines through the field of view. Within the granular layer, we selected for analysis only neuronal somata containing the initial segment of dendrites and axons, complete from end-to-end across the image stack so that all mitochondria within a given soma were reconstructed (Figure S2). In total, we analyzed 42 myelinated axons (DG, n = 21; CA1, n = 21), 42 dendrites (DG, n = 21; CA1, n = 21), and 33 somata (DG, n = 19; CA1, n = 14). From these compartments, a total of 1,573 (DG) and 3,892 (CA1) mitochondria were analyzed (see Tables S1 and S2 for details by subcompartment). Figures 1D and 1E illustrate typical images of reconstructed CA1 axon, dendrite, and soma mitochondria.

### Comparison of mitochondrial size and morphology across subcellular compartments

**Figure 2.**
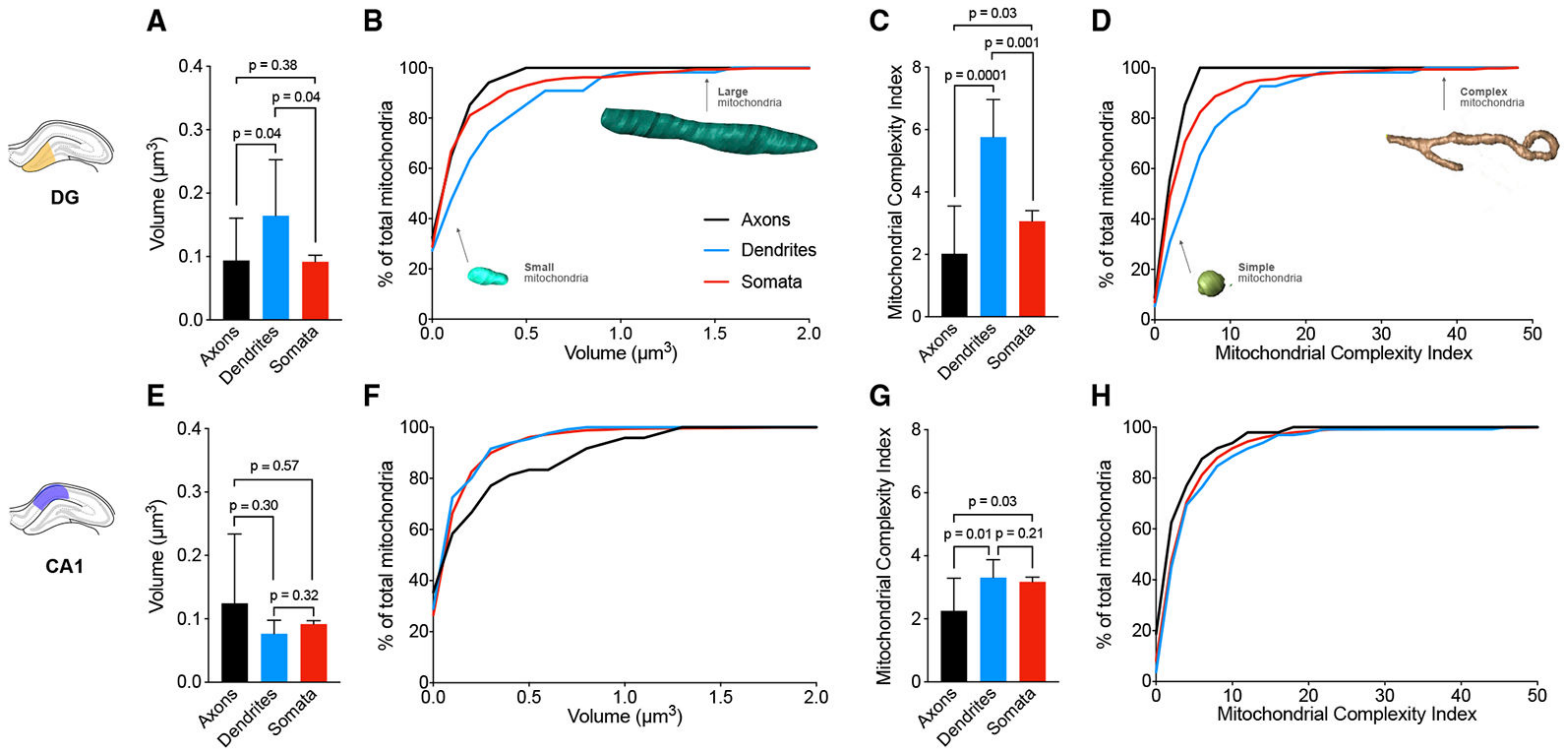
Differences in mitochondrial morphology between axons, dendrites, and somata (A and B) Average DG mitochondrial volume (A) and cumulative frequency distribution (B) in axons, dendrites, and somata. (C and D) Same as (A) and (B) for mitochondrial complexity index (MCI). Example mitochondria at different portions of the frequency distributions are shown for reference. n = 34 axonal, n = 55 dendritic, and n = 471 somatic mitochondria, across three axons, dendrites, and somata. (E and F) Average CA1 mitochondrial volume (E) and cumulative frequency distribution (F) in axons, dendrites, and somata. (G and H) Same as (E) and (F) but for MCI. n = 48 axonal, n = 131 dendritic, and n = 1,588 somatic mitochondria, across three axons, three dendrites, and two somata. Data are presented as median with 95% confidence interval (CI). Kruskal-Wallis test followed by post hoc tests using the two-stage step-up method of Benjamini, Krieger, and Yekutieli to correct for multiple comparisons (p < 0.05, q < 0.05).

We first compared mitochondrial size in axons, dendrites, and somata of both the DG and CA1 in young mice (Figures 2A-2H; Video S1). In DG, dendritic mitochondria of granule cells (Video S4) were on average 116% larger (mean volume = 0.27 μm^3^) than axonal mitochondria in afferent axons (0.12 μm^3^, p = 0.04, Video S3), and 42% larger than somatic mitochondria (mean volume = 0.19 μm^3^, p = 0.04, Video S5). A small proportion of dendritic (9.1%) and somatic (3.8%) mitochondria were exceptionally large (>1 μm^3^), whereas no axonal mitochondria (0%) reached 1 μm^3^ (Figures 2A and 2B), highlighting gross differences in the distribution of mitochondrial volume between subcellular compartments. Mitochondrial volume heterogeneity was evident at multiple levels of analysis in axons and dendrites (Figures S3A-S3H).

To quantify mitochondrial morphological complexity, we next applied the mitochondrial complexity index (MCI) (Vincent et al., 2019). Based on this metric, in the DG, dendritic mitochondria were the most complex (mean MCI = 6.9) of the three compartments, with an average MCI 143.3% greater than that of axonal mitochondria (mean MCI = 2.8, p = 0.0001), and also 41.7% greater than that of somatic mitochondria (mean MCI = 4.8, p = 0.001, Figures 2C and 2D).

Interestingly, in CA1, dendritic mitochondria of CA1 pyramidal cells (Video S7) tended to be smaller (mean volume = 0.14 μm^3^, p = 0.30) than axonal mitochondria from afferent axons, which were on average 84.7% larger (mean volume = 0.27 μm^3^, Video S6). No difference was observed in dendritic compared to somatic mitochondria (mean volume = 0.15 μm^3^, p = 0.32, Video S8). Axonal mitochondria were on average 67.5% larger than somatic mitochondria (p = 0.57), with a higher proportion (12.5%) of largest mitochondria (volume > 0.75 μm^3^) compared to somatic mitochondria (1.88%) (Figures 2E and 2F). Similar to the DG, despite their larger size, axonal mitochondria were the least complex (mean MCI = 3.5) compared to either dendritic mitochondria (mean MCI = 5.2, p = 0.01) or those in the somata (mean MCI = 4.7, p = 0.03, Figures 2G and 2H).

These data confirmed previous findings that, in both CA1 and DG, axons harbor simpler mitochondria than dendrites. Moreover, mitochondrial volume and complexity in the somata of DG and CA1 regions were intermediate relative to axons and dendrites.

### Comparison of mitochondrial morphology between DG and CA1

**Figure 3.**
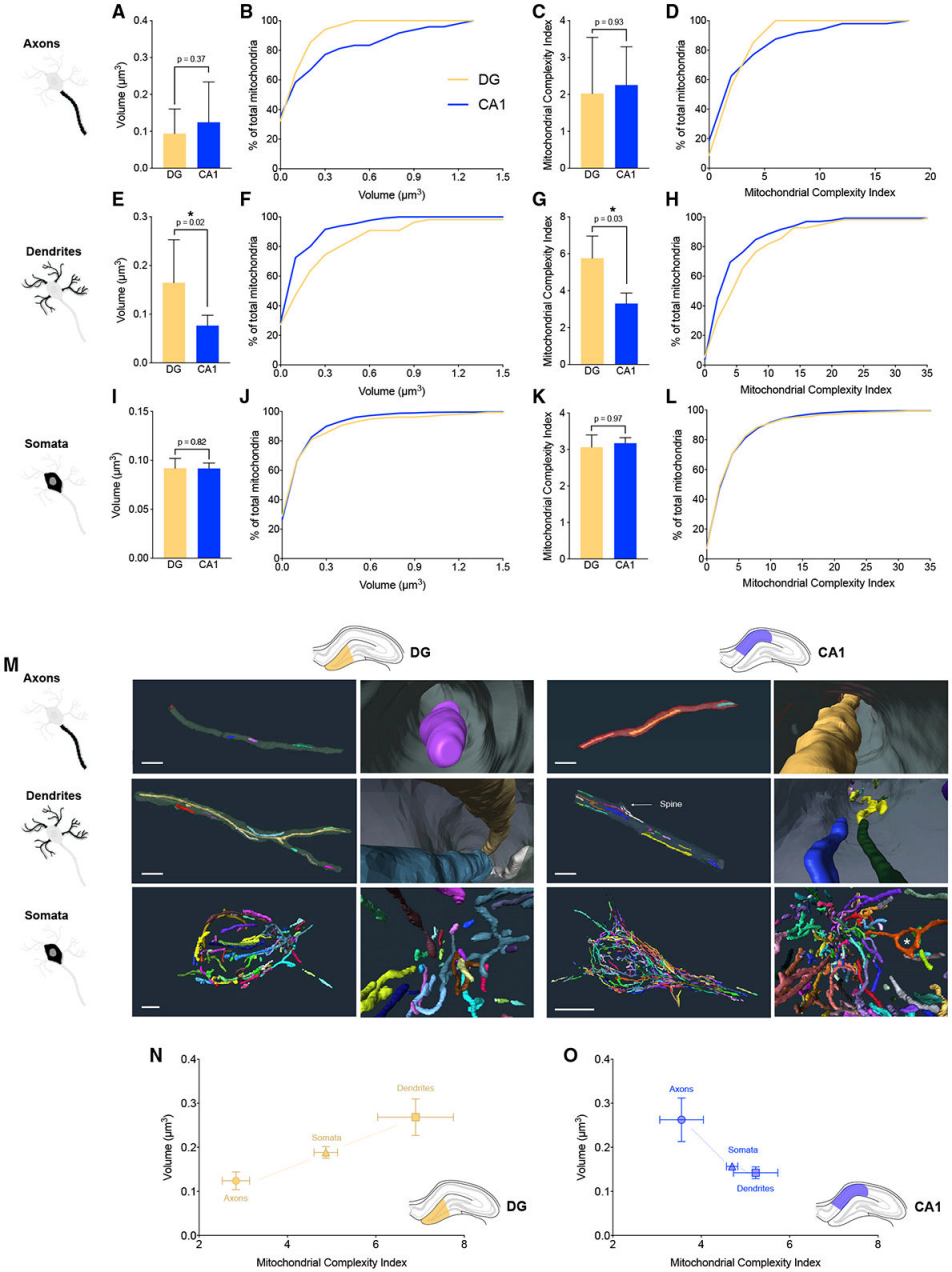
Differences in mitochondrial morphology between DG and CA1 and 3D reconstructions to compare axonal, dendritic, and somatic mitochondria (A–D) Axonal mitochondrial volume (A and B) and MCI (C and D) in DG and CA1. (E–H) Dendritic mitochondrial volume (E and F) and MCI (G and H) in DG and CA1. (I–L) Somata mitochondrial volume (I and J) and MCI (K and L) in DG and CA1. (M) Reconstruction of mitochondrial network in axons, dendrites, and somata of the mouse DG and CA1. Each mitochondrion is a different color. Right panels are higher magnifications of the left panels. *Donut mitochondrion. Scale bars, 2 μm. (N and O) Mitotype graphical representation of the morphological complexity (x axis) and volume (y axis) of mitochondria (mean ± SEM) in three different compartments imaged from the DG (N) and CA1 (O). Only the dendritic mitochondria showed significant differences between the two regions, with larger and more complex mitochondria in the DG than in the CA1. DG: n = 34 axonal, n = 55 dendritic, and n = 471 somatic mitochondria, across three axons, dendrites, and somata per mouse. CA-1: n = 48 in axonal, n = 131 dendritic, and n = 1,588 somatic mitochondria, across three axons, dendrites, and two somata per mouse. Data are presented as median with 95% CI. Two-tailed Mann-Whitney non-parametric tests (*p < 0.05).

Following the comparison of mitochondrial morphology between subcellular compartments, we directly compared morphological parameters between DG and CA1 for each subcellular compartment. Within the axons in the CA1, mitochondria tended to be on average 112% larger (not significant [N.S.]) than in the DG, mostly driven by the presence of very large mitochondria in CA1 that are not observed in the DG (Figures 3A and 3B). The average MCI did not show a significant difference in axonal mitochondria between DG and CA1 (p = 0.93), although CA1 axons also had 12.5% more highly complex mitochondria with MCI > 7 not observed (0%) in the DG (Figures 3C and 3D). Dendritic mitochondria in the DG were both larger (89%, p = 0.02) and more complex (MCI 31.9%, p = 0.03) than in the CA1 (Figures 3G and 3H). For somata, both mitochondrial volume (p = 0.82) and MCI (p = 0.97) were similar across DG and CA1 (Figures 3I-3L).

These results document the existence of notable differences in dendritic mitochondria and projecting axons between DG and CA1 (Figure 3M). In contrast, the soma of DG granule cells and CA1 pyramidal cells have mitochondria with similar morphological characteristics. This could be an important observation that reflects the function of presynaptic versus postsynaptic dynamics in these two regions. In brief, axons exhibited a straight cell surface with more small simple mitochondria in DG and larger simple mitochondria in CA1. Dendrites show large complex mitochondria in DG and smaller complex mitochondria in CA1. Somata of pyramidal neurons in both CA1 and DG exhibit a population of mitochondria that are a morphological intermediary between axonal and dendritic mitochondria. Plotting the mean volume and MCI together on a mitochondrial phenotype (i.e., mitotype) graph for DG (Figure 3N) and CA1 (Figure 3O) highlights the relative similarity of somatic and dendritic mitochondria in CA1.

The subcellular topology of mitochondria in both areas also differed between subcellular compartments. Whereas axonal mitochondria tended to be either distant from each other or positioned in succession along the axon lumen, in dendrites the elongated mitochondria often overlapped with each other. For example, across the diameter of single dendrites, we observed more mitochondria, particularly in proximity to dendritic spines, likely to meet regional energy demands and to contribute to local Ca^2+^ buffering (see CA1 in Figure 3M). In the soma only, we noted the existence of donut mitochondria (asterisk in Figure 3O, CA1), which reflect mitochondria that loop and fuse with themselves (Long et al., 2015). As partially reflected in the MCI, somatic mitochondria harbor substantially more convoluted shapes, likely reflecting a combination of both larger available volume and more complex cytoskeletal organization relative to the filiform axons and dendrites and potentially different biochemical functions.

### Effect of aging on axonal, dendritic, and somatic mitochondria in DG and CA1

**Figure 4.**
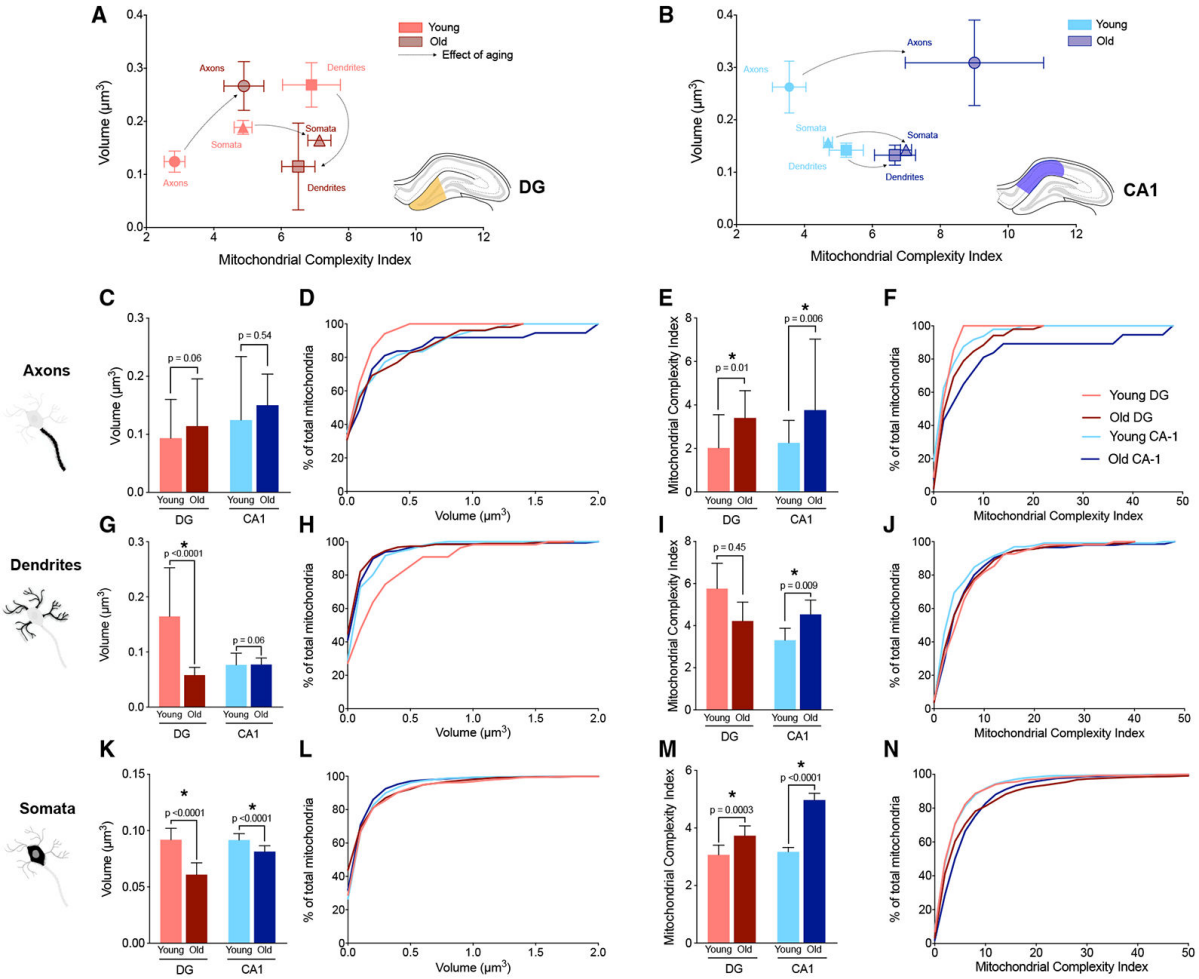
Effect of aging on mitochondrial morphology in DG and CA1 mitochondria (A and B) Mitotypes illustrating the difference between aged and young mitochondria (mean ± SEM) across compartments in the DG (A) and CA1 (B). (C–F) Axonal mitochondrial volume (C and D) and MCI (E and F) comparison between young and old animals. MCI were significantly increased with age in both regions. (G–J) Dendritic mitochondrial volume (G and H) and MCI (I and J) comparison between young and old animals. With aging, dendritic mitochondria in the old DG were clearly smaller (mean volume = 0.11 μm^3^) than mitochondria in the young DG. The MCI increased with age only in CA1. (K–N) Somatic mitochondrial volume (K and L) and MCI (M and N) comparison between young and old animals. With aging, the mitochondrial volume decreased significantly in both regions; however, the complexity increased significantly with age, giving smaller mitochondria but complexity with age. Young DG: n = 34 axonal, n = 55 dendritic, and n = 471 somatic mitochondria. Old DG: n = 52 axonal, n = 184 dendritic, and n = 777 somatic mitochondria. Young CA1: n = 48 axonal, n = 131 dendritic, and n = 1,588 somatic mitochondria. Old CA1: n = 37 axonal, n = 146 dendritic, and n = 1,930 somatic mitochondria. n = 3 axons, dendrites, and somata analyzed for each region (DG and CA1), in young and old (total 12 of each). Data are presented as median with 95% CI. Kruskal-Wallis test followed by post hoc tests using the two -stage step-up method of Benjamini, Krieger, and Yekutieli to correct for multiple comparisons (* p<0.05, q<0.05).

To investigate the effect of mitochondrial aging in the hippocampal DG and CA1 mitochondria, we compared morphological parameters of the young mice described above to 18-month-old syngeneic mice. We used the same mitotype graph as above to highlight the regional differences in mitochondrial volume and complexity with aging. The general effect of aging on mitochondrial volume and MCI is shown, separately for the DG and CA1, by the dashed arrows (Figures 4A and 4B; Figures S3I-S3J). As in young mice, mitochondria in axons, dendrites, and somata in old animals generally had distinct mitochondrial morphologies (Video S2).

In the DG of old mice, contrary to young animals, dendritic mitochondria were on average 56.8% smaller (0.11 μm^3^, Video S10) than axonal mitochondria (0.27 μm^3^, p = 0.002, Video S9) and 29.9% smaller than somatic mitochondria (0.16 μm^3^, p = 0.12, Video S11) (Figure S3I). Axonal mitochondria were 62.5% larger than somatic mitochondria (p = 0.01) (Figure 4A; Figure S3I). Old somatic mitochondria tended to be on average the most complex (Figure 4A; Figure S3I).

Interestingly, in the CA1, dendritic mitochondria were also the smallest, on average 57% smaller (0.13 μm^3^) than in axons (0.31 μm^3^, p = 0.01) and 6.9% smaller than in the soma (0.14, p = 0.11, Figure 4B; Figure S3J, Videos S12-S14). No differences were seen in complexity, but in contrast to young animals, the old axonal mitochondria were larger than both dendritic (35%) and somatic mitochondria (28.8%) (Figure 4B; Figure S3J, Videos S12-S14).

Thus, in old animals, axonal mitochondria were the largest in both DG and CA1, but were most complex in the CA1, pointing to the differential effects of aging between hippocampal regions. Furthermore, aging reversed the larger size of dendritic versus axonal mitochondria in DG relative to CA1.

### Effect of aging on DG and CA1 mitochondria

The average volume of axonal mitochondria did not show any significant volume differences between young and aged mice, although old animals showed a greater proportion of larger mitochondria (>0.5 μm^3^, 17.3%) than young animals (0%, Figures 4C and 4D). Within the DG, old axonal mitochondria were 114.8% more complex than in young animals (p = 0.01, Figures 4E and 4F). This was also due to a greater proportion of highly complex mitochondria (MCI > 7) in old axons (21.5%) and a complete absence of complex mitochondria in young axons (Figures 4E and 4F). Second, dendritic mitochondria of old animals were 58.2% smaller than in young animals (p < 0.0001) (Figures 4G and 4H). However, in contrast to axons, aging did not affect dendritic mitochondrial complexity (Figures 4I and 4J). Third, old somatic mitochondria were 13.1% smaller (p < 0.0001, Figure 4K and 4L), but 46.5% more complex (p = 0.0003) in the old than in the young hippocampus (Figures 4M and 4N).

In the CA1 region, aging did not affect the volume of axonal mitochondria (p = 0.54, Figures 4C and 4D) but was associated with greater complexity (mean MCI = 9, p = 0.006) than in young animals (mean MCI = 3.5, Figures 4E and 4F). Similarly, aging did not significantly affect dendritic mitochondrial volume (p = 0.06, Figures 4G and 4H) but mitochondrial complexity was 27.5% higher (p = 0.009, Figures 4I and 4J). Finally, in contrast to axonal and dendritic mitochondria, the somatic mitochondria of old animals were on average 10% smaller (mean volume = 0.14 μm^3^, p < 0.0001) than those of young mice (mean volume = 0.16 μm^3^, Figures 4K and 4L) and also more complex (mean MCI = 7.0, p < 0.0001) than young CA1 somatic mitochondria (mean MCI = 4.7, Figures 4M and 4N).

These data indicate that aging leads to a general increase in mitochondrial morphological complexity within multiple neuronal subcompartments from both DG and CA1, particularly incoming axons. However, compared to the CA1, DG mitochondria are in most cases more severely impacted by aging, exhibiting larger effect sizes for mitochondrial volume changes, with larger axonal mitochondria and smaller dendritic and somatic mitochondria.

### Effect of aging on axon and dendrite size and mitochondrial volume density

To understand whether the cell compartment volume was related to age-related changes in mitochondrial morphology, we also compared the volume and diameter of axons and dendrites (Figures S4A and S4B). DGaxons volume did not show any difference with age; however, dendrites were 138% larger in old versus young DG (p = 0.04). Similarly, there was no age-related difference for DGaxon diameter; however, dendrites were 30% larger in older animals (p = 0.03). In the CA1 region, aging did not affect the volume or diameter of axons or dendrites (Figures S4A and S4B).

We then broadened our analysis beyond the morphological properties of mitochondria to examine their abundance (i.e., volume density) in each subcellular compartment. Mitochondrial volume density (MVD) is calculated as the proportion of intracellular volume occupied by mitochondria. In young brains, axonal MVD was 153.9% higher in the CA1 than in the DG region (p = 0.009), but the opposite was observed in dendrites where the CA1 had 45.1% lower MVD than the DG (p = 0.03, Figure S4C). This difference points toward potentially important differences related to DG granule and CA1 pyramidal neuron activity or biochemical needs for dendrites, and different projection sources of the incoming axons.

In relation to aging, MVD in old CA1 axons was 45.6% lower than in young brains (p = 0.009, Figure S4C). The effect tended to be the opposite in DG axons, where old DG axons had 80.9% higher MVD than young axons (p = 0.07). In old dendrites of both hippocampal regions MVD tended to be 30% (DG) and 17% (CA1) lower (N.S.) with aging, although we noted substantial heterogeneity and the existence of a subgroup of dendrites with remarkably high MVD (Figure S4C).

### Effect of aging on soma size and mitochondrial volume density

**Figure 5.**
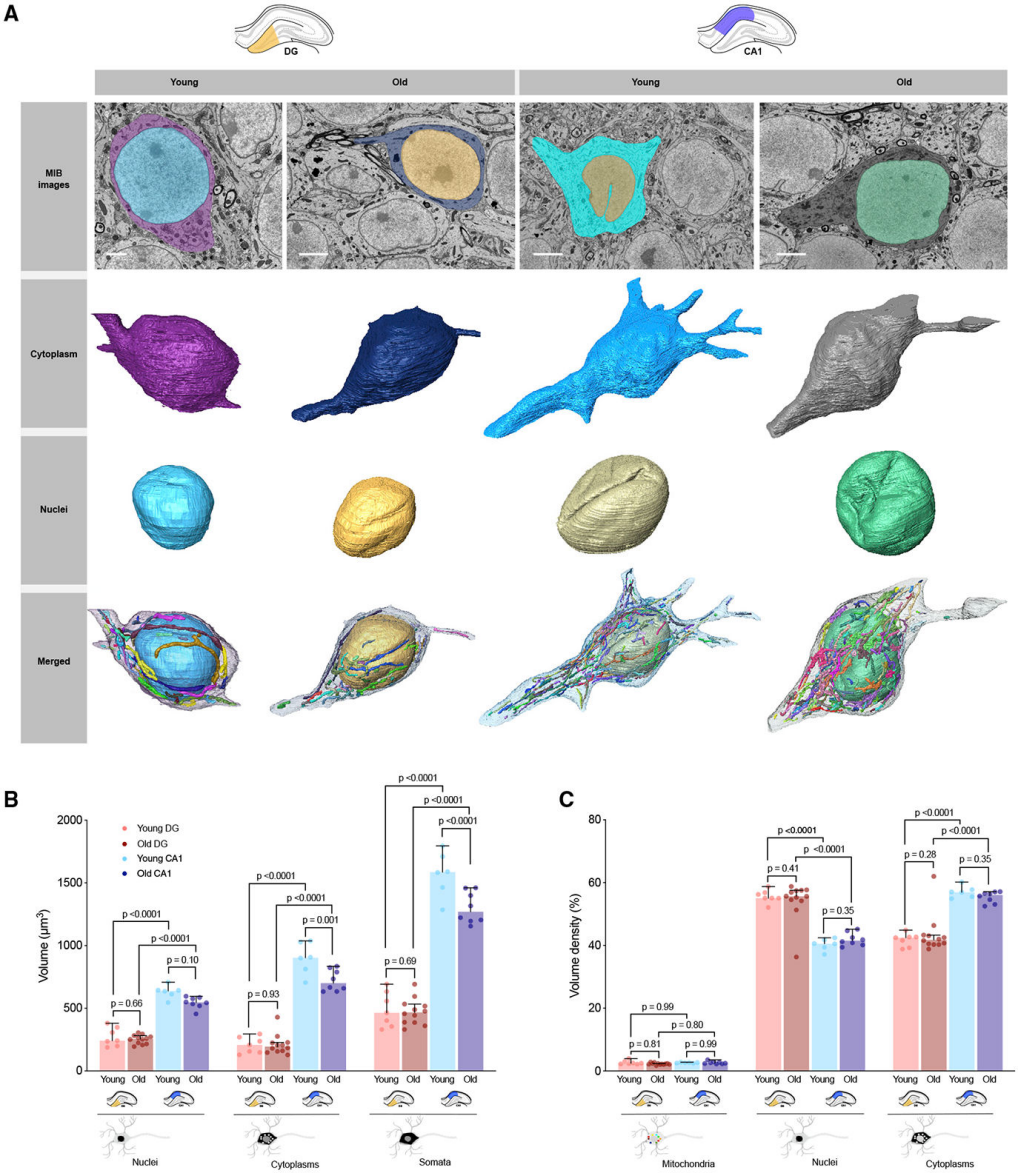
Effect of aging on subcellular compartments volume within the DG and CA1. (A) Representative 2D electron micrograph from the SBF-SEM datasets (top) and 3D reconstructions of the cell membrane bounding the cytoplasmic space, the nuclei, and all surrounding mitochondria. The cytoplasmic volume is calculated without the mitochondria and nucleus. Representative images of somata from both young and old mice in the DG and CA1 are shown. Scale bars, 3 μm. (B) Volume of young and old nuclei, cytoplasms, and somata in the DG and CA1. (C) Volume of somatic mitochondria, nuclei, and cytoplasms expressed relative to the total cellular volume, representing volume density and their differences between the young and old DG and CA1. DG: n = 7 young and n = 12 old somata; CA1: n = 6 young and n = 8 old somata. Data are presented as median with 95% CI. Kruskal-Wallis test followed by post hoc tests using the two-stage step-up method of Benjamini, Krieger, and Yekutieli to correct for multiple comparisons (p < 0.05, q < 0.05).

To assess how changes in mitochondria with aging relate to overall changes in cell morphology, we quantified the total soma volume of DG granule and CA1 pyramidal neurons in young and old animals. The volume of the nucleus was also quantified to derive cytoplasmic volume (Figure 5A). In agreement with readily observed differences in the size of principal cell types in the regions, we observed marked differences in somatic volume between the DG and CA1. In the same animals, compared to the DG, the CA1 pyramidal neurons had larger nuclei (132.3%), cytoplasmic space (335.7%), and total somatic volume (220.3%, ps < 0.0001) (Figure 5B). Moreover, whereas 55.6% of the cytoplasmic volume is occupied by the nucleus in the DG, this proportion is 40.3% (difference of −15.3%) in CA1 neurons (Figure 5C).

In relation to aging, whereas DG granule neuron somatic, nuclear, and cytoplasmic volume were not different between old and young animals, CA1 neurons showed atrophied somata (−17.2%, p < 0.0001) and cytoplasmic volume (without nucleus and mitochondria) (−19.7%, p = 0.001, Figure 5B) in old animals. Despite the apparent atrophy of CA1 somata, the volume of the nuclei was more moderately affected with aging (−13.6%, p = 0.10, N.S.), indicating that old pyramidal cells have larger nuclei relative to the cell body volume.

We also computed MVD in somata, as in axons and dendrites, which showed that MVD in relatively distinct DG granule and CA1 pyramidal cells were similar. MVD also did not differ between old and young hippocampi. The percentage of total cellular volume occupied by the nucleus and cytoplasm also did not show any differences with aging (Figure 5C). Taken together, these data indicate that although the aging CA1 pyramidal neurons undergo substantial atrophy, the relative proportions of different cell compartment mitochondria, including MVD, are relatively unchanged in the aging mouse brain (Figure S5).

## DISCUSSION

Using SBF-SEM 3D electron microscopy, we have quantified the distribution and heterogeneity of mitochondrial morphology in different cellular appendages of hippocampal DG and CA1 regions in young and aged mice. Using the strictly laminated structure of these regions, we could compare mitochondrial features within specific subcellular compartments in these regions. Our analyses confirmed the structural specificity between axonal, dendritic, and somatic mitochondria and provided high-resolution quantitative information about both mitochondrial volume and morphological complexity across both DG and CA1 regions. Moreover, our data highlight age-related recalibrations in mitochondrial morphology and soma characteristics, which differ by subcellular compartment and between DG and CA1 regions.

### Mitochondrial morphological specificity within neuronal subcompartments

In neurons, mitochondria are transported to sites with high energy demand or specific biochemical requirements, such as growth cones, pre- and post-synaptic elements, and nodes of Ranvier (Hollenbeck and Saxton, 2005). Mitochondrial morphology and function (i.e., morpho-function) are interlinked in mammalian cells (Bulthuis et al., 2019), and changes in mitochondrial morphology impact neuronal mitochondrial Ca^2+^ uptake and synaptic transmission (Lewis et al., 2018), positioning mitochondria as neuromodulators.

Our data confirm that mitochondria in the mouse brain exhibit distinct morphology in axons and dendrites and add quantitative information about these differences. Extending previous reports (Popov et al., 2005), we find that across both young CA1 and DG, axonal mitochondria were simple compared to the more complex dendritic mitochondria. This simple morphology is essential for axonal mitochondrial trafficking (Li et al., 2004), as punctate mitochondria are more easily transported along microtubules through the narrow axon lumen than tubular organelles. The function of mitochondria also differs between axonal and dendritic mitochondria, with dendritic mitochondria maintaining a greater membrane potential than axonal mitochondria (Overly et al., 1996), in keeping with the notion that elongated mitochondria may have greater coupling efficiency (Gomes et al., 2011). We also find that somatic mitochondrial volume is intermediate relative to the other neuronal compartments in DG and show relative similarity with dendritic mitochondria in CA1.

The morphological specialization of mitochondria between different compartments of a given cell type is not unique to neurons. In skeletal muscle, mitochondria in the same cell also have different morphologies, with differences of ~1- to 1.5-fold in humans (Vincent et al., 2019) and ≥ 1-fold in the mouse soleus (Picard et al., 2013). In comparison, in neurons, we find differences of 1- to 2-fold between axons and dendrites, indicating that morphological specialization may be particularly pronounced in neurons. Thus, we speculate that cell-level complexity, such as the highly specialized axons and dendrites of neurons or the subsarcolemmal and intermyofibrillar compartments delimited by the contractile apparatus in muscle, may contribute to generating biochemically and energetically distinct cellular compartments, which in turn drive the establishment of specialized size, morphology, and function among resident mitochondria.

### Aging is associated with selective changes in mitochondrial morphology in the DG

Our data show that the different hippocampal regions exhibit different degrees of age-related changes. Previous work has shown that the DG undergoes greater age-related changes relative to the CA1, perhaps related to the capacity of the DG region for adult neurogenesis, which declines with age (Pawluski et al., 2009). Some of the impairment in neurogenesis observed with aging could be related to a decline in respiratory chain function (Navarro et al., 2011; Holper et al., 2019). For example, ablation of the mitochondrial transcription factor A (Tfam) in quiescent neural stem cells impaired mitochondrial function and neurogenic potential (Beckervordersandforth et al., 2017). Furthermore, Braidy et al. (2014) found reduced activities of mitochondrial complex I–IV in aged rodent brains. These mitochondrial impairments are the direct consequence of a decrease in electron transfer rate (Navarro and Boveris, 2004).

Based on the age-related decline in DG function compared to the CA1, we initially hypothesized that mitochondrial morphology might also be preferentially altered by aging in the DG than CA1. Molecularly, previous work showed that different hippocampal regions have distinct gene and protein expression patterns in mice (Farris et al., 2019), and Ca^2+^-binding proteins are more abundant in the DG than CA1 (Frantz and Tobin, 1994). In this study, we found that changes in mitochondrial morphology occur in both areas but they were indeed more pronounced in the DG. In the CA1, mitochondrial complexity increased with age in all subcellular compartments. The MCI also generally increased with age within the DG. A similar phenotype of increased mitochondrial complexity with aging was also observed in aging mouse skeletal muscle (Leduc-Gaudet et al., 2015), postulated to reflect compensatory hyperfusion response to stress, previously reported in cellular systems (Shutt and McBride, 2013).

### Conclusions

While it is accepted that mitochondria functionally and morphologically specialize to meet the metabolic and biochemical demands of their local environment, the morphological characteristics of mitochondria in different neuronal appendages of the aging DG and CA1 had not been defined. With our study, we have confirmed that mitochondrial morphology differs between subcellular location, and that axonal and dendritic mitochondria are generally morphological opposites in young healthy animals. Quantification of mitochondrial morphology in young and old animals brains showed particularly marked differences in the DG, consistent with previous evidence indicating that the DG may be more vulnerable to the effects of aging. Taken together, our findings lay the foundation to investigate potential mechanistic links between mitochondrial morphology and neuronal function, together with circuit-level function and related behaviors.

## STAR★METHODS

### RESOURCE AVAILABILITY

#### Lead contact

Further information and requests for resources and reagents should be directed to and will be fulfilled by the lead contact, Martin Picard (martin.picard@columbia.edu).

#### Materials availability

This study did not generate new unique reagents.

#### Data and code availability

All data reported in this paper will be shared by the lead contact upon request.

This paper does not report original code.

Any additional information required to reanalyze the data reported in this paper is available from the lead contact upon request.

### EXPERIMENTAL MODELS AND SUBJECT DETAILS

#### Animals and tissue collection

All experiments involving animals were approved by the Institutional Animal Care and Use Committee of Columbia University Medical Center (IACUC) and were performed in accordance with relevant guidelines and regulations. Mice were maintained under standard conditions, housed in a specific pathogen–free animal house under a 12/12-hour light/dark cycle and were provided food and water *ad libitum*. All wild-type mice C57Bl6J (female) were purchased from Jackson Laboratories (RRID:IMSR_JAX:000664). Young mice were 4 months (n = 3) and old mice were 18 months (n = 4). Animals were anesthetized with a mixture of ketamine/xylazine (100/20mg/kg) via intraperitoneal injection immediately before fixation.

### METHOD DETAILS

#### Serial block face scanning electron microscopy (SBF-SEM)

Antesthetized mice were transcardially perfused using buffered 4% glutaraldehyde with 2% paraformaldehyde in in 0.1M cacodylate buffer. The brain was resected and kept in fixative until processing. Thin brain sections (200 μm) were then obtained using a vibratome and stored in fixative. Small segments that included the dorsal DG and CA1 (bregma = 1.64mm) were dissected with a scalpel blade and processed using a heavy metal protocol adapted from (Deerinck et al., 2010). Samples were washed in 0.1M sodium cacodylate pH7.4 followed by an immersion in 3% potassium ferrocyanide with 2% osmium tetroxide for 1 hour. The samples were put into a contrast enhancer thiocarbohydrazide 0.1% for 20 min, and then 2% osmium tetroxide for 30 min, and finally placed into 1% uranyl acetate overnight at 4°C. Between each step, all samples were washed in several changes of ddH_2_O. The following day, samples were immersed in lead aspartate solution (0.12 g of lead nitrate in 20 mL aspartic acid) for 30 min at 60°C. Samples were then dehydrated in a graded series of acetone from 25% to 100% and then impregnated in increasing concentrations of Taab 812 hard resin in acetone with several changes of 100% resin. The samples were embedded in 100% fresh resin and left to polymerize at 60°C for a minimum of 36 hours. After polymerization, the resin blocks were trimmed and the regions of interest (ROIs, see Figure S1) were identified on thin slices by light microscopy.

Following trimming, samples were placed into a Zeiss Sigma SEM incorporating the Gatan 3view system (Gatan inc., Abingdon, UK) for SBFSEM, which allows sectioning of the block and the collection of serial images in the z-plane. For each mouse, 4 regions of interest (ROIs) in the dentate gyrus (DG, 2 ROIs) and in the cornu-ammonis region 1 (CA1, 2 ROIs) (Figure S1). These ROIs were sectioned and captured in a series of images (400 images per stack) at 50 nm sectioning thickness covering a volume of 40 μm x 40 μm x 20 μm. Pixel density was 4,000 × 4,000 (x, y), for a final pixel size of 10nm x 10nm x 50nm (x, y, z). The serial images acquired were handled and processed for segmentation with MIB (Microscopy Image Browser, Helsinki) (Belevich et al., 2016).

#### 3D reconstructions and quantitative analysis

Image stacks were exported and analyzed. To prevent potential bias related to the effects of age and regional variation, reconstructions and data analysis were performed blinded to age group. For each reconstructed mitochondrion, the total volume and surface area was quantified using AMIRA, and used to compute morphological complexity. Somata, nuclei, myelinated axons, dendrites and mitochondria were tracked in all three dimensions for reconstructions with MIB. Each subcellular compartment and their mitochondria were manually traced in MIB using the ‘brush’ drawing tool (manual segmentation) and were excluded if they were not completely within the ROI. Each cellular structure was assigned a different color during the segmentation process. The models were exported to AMIRA, and for each completely reconstructed structure, the total volume and surface area were extracted. The Mitochondrial Complexity Index (MCI) was calculated using the formula detailed in Vincent et al. (2019) and provided below:

$$MCI={\left(\frac{\left(SA^{1.5}\right)}{4\pi V}\right)}^{2}=\frac{SA^{3}}{16\pi^{2}V^{2}}$$

This equation is a three-dimensional equivalent to form factor, to assess mitochondrial morphological complexity.

The quantification of the axon and dendrite diameters was done by using MIB “measure the length” tool. The diameter at 10 equally spaced points along each structure and the average of these 10 measurements were used as the diameter of the axon or dendrite.

### QUANTIFICATION AND STATISTICAL ANALYSIS

Differences in average values of shape descriptions used to assess effect of aging on mitochondrial morphology were analyzed using a Kruskal-Wallis test followed by post hoc tests using the two-stage step up method of Benjamini, Krieger, and Yekutieli to correct for multiple comparisons (p < 0.05, q < 0.05) (Figures 2, 5, S3, and S4).

Differences in average values of shape descriptions used to assess mitochondrial morphology for each single sub-compartment per regions were statistically analyzed using nonparametric Mann–Whitney test, because normalization was not effective (Figures 3, 4, and S5). Differences between each cell and nuclei volume were statistically analyzed using a Two–Way ANOVA followed by post hoc tests using the two-stage step up method of Benjamini, Krieger, and Yekutieli to correct for multiple comparisons (p < 0.05, q < 0.05). The exact value of n are indicated in figure legends. The statistical tests used and p values are indicated in figures. All statistical analyses were performed using the Prism 8 software (GraphPad Software, San Diego, CA, USA).

#### Limitations

All subcellular compartments do not necessarily correspond to the same cell for both brain regions. The axons within the DG and CA1 regions originate from different brain locations. The DG and CA1 molecular layers received inputs from medial-entorhinal cortex and from the CA3, respectively. Furthermore, to achieve the segmentation of the full somata without cropping any mitochondria, the initial segment of dendrites and axons (axon hillock) were included in the reconstructions, as shown in Figure 5A.

## Supplementary Material

1

2

3

4

5

6

7

9

10

11

12

13

14

15

16

## Figures and Tables

**Table:** KEY RESOURCES TABLE

REAGENT or RESOURCE	SOURCE	IDENTIFIER
Chemicals, peptides, and recombinant proteins		
Acetone	Fisher	Cat# A060617
Aluminum pin	Taab	Cat# G312
Aspartic acid	Sigma Aldrich	Cat# A9256
Cacodylate buffer	Agar Scientific	Cat# R1104
Copper grids	Gilder grids	Cat# GA1500-C3
Epoxy resin	Taab	Cat# T030
Glutaraldehyde	Taab	Cat# G003
Gold coating	Agar Scientific	Cat# B7370
Lead Citrate	Sigma-Aldrich	Cat# 228621
Potassium ferrocyanide	Sigma-Aldrich	Cat# 244023
Osmium tetroxide	Agar Scientific	Cat# AGR1024
Thiocarbohydrazide	Sigma-Aldrich	Cat# 223220
Toluidine blue	Taab	Cat# SD211
Silver glue	Agar Scientific	Cat# G3648
Uranyl acetate	Agar Scientific	Cat# AGR1260A
Experimental models: Organisms/strains		
Mice C57Bl6J (female)	Jackson lab	RRID: IMSR_JAX:000664
Software and algorithms		
MIB	Ilya Belevich: University of Helsinki	http://mib.helsinki.fi/
Amira	Thermo Fisher Scientific	https://www.thermofisher.com/global/en/home/industrial/electron-microscopy/electron-microscopy-instruments-workflow-solutions/3d-visualization-analysis-software/amira-life-sciences-biomedical.html
		RRID: SCR_007353
Digital Micrograph	Gatan	https://www.gatan.com/products/tem-analysis/gatan-microscopy-suite-software
Prism 9	GraphPad	https://www.graphpad.com/scientific-software/prism/
Other		
Serial block face SEM	Gatan	3view
SEM	Zeiss	Sigma

## Highlights

- Neuronal dendrites, somata, and myelinated axons have different mitochondrial morphology
- The mouse hippocampal CA1 and DG regions have different mitochondrial populations
- Aging preferentially affects the morphology of DG mitochondria
- Aging increases the size of DG dendrites and causes neuronal somata atrophy in CA1
