# Supplementary material for: 3D neuronal mitochondrial morphology in axons, dendrites, and somata of the aging mouse hippocampus
Source: Cell Rep. Author manuscript; Available in PMC 2021 Sep 7. (PMC8423436; doi:10.1016/j.celrep.2021.109509)
Supplement: 1 [file NIHMS1732407-supplement-1.pdf]

Cell Reports, Volume *36*

Supplemental information

3D neuronal mitochondrial morphology in axons, dendrites, and somata of the aging mouse hippocampus

Julie Faitg, Clay Lacefield, Tracey Davey, Kathryn White, Ross Laws, Stylianos Kosmidis, Amy K. Reeve, Eric R. Kandel, Amy E. Vincent, and Martin Picard

	Axons		Dendrites		Somata	
	Young	Old	Young	Old	Young	Old
DG	34	52	55	184	471	777
CA-1	48	37	131	146	1588	1942

Table S1. Total of Mitochondrial count across neurons sub-cellular compartments in DG and CA1 regions and detailed per mouse. Related to Figure 2 and 4.

	Axons		Dendrites		Somata	
**DG**	**Young**	**Old**	**Young**	**Old**	**Young**	**Old**
**M1**	6 / 2 / 4		3 / 4 / 6		37 / 78 / 54	
**M2**		5 / 2 / 2		3 / 1 / 3		57 / 71 / 77
**M3**	6 / 4 / 4		5 / 3 / 2		70 / 41	
**M4**		6 / 2 / 2		10 / 16 / 17		80 / 64 / 73
**M5**	4 / 2 / 2		11 / 12 / 9		45 / 146	
**M6**		2 / 3 / 3		13 / 24 / 24		62 / 65 / 65
**M7**		2 / 5 / 16		15 / 33 / 35		76 / 52/ 35
**CA1**	**Young**	**Old**	**Young**	**Old**	**Young**	**Old**
**M1**	1 / 3 / 4		17 / 6 / 10		264 / 262	
**M2**		3 / 6 / 2		5 / 13 / 6		173 / 149
**M3**	24 / 5 / 3		13 / 21 / 9		229 / 266	
**M4**		2 / 3 / 1		20 / 20 / 30		239 / 214
**M5**	2 / 2 / 4		22 / 21 / 12		256 / 311	
**M6**		2 / 6 / 3		7 / 4 / 8		290 / 309
**M7**		0 / 2 / 0		6 / 9 / 18		253 / 315

Table S2. Number of mitochondria analyzed for each subcellular compartment (3 axons, 3 dendrites, 2 or 3 somata) in DG and CA1 regions, detailed per mouse. Related to Figure 2 and 4.

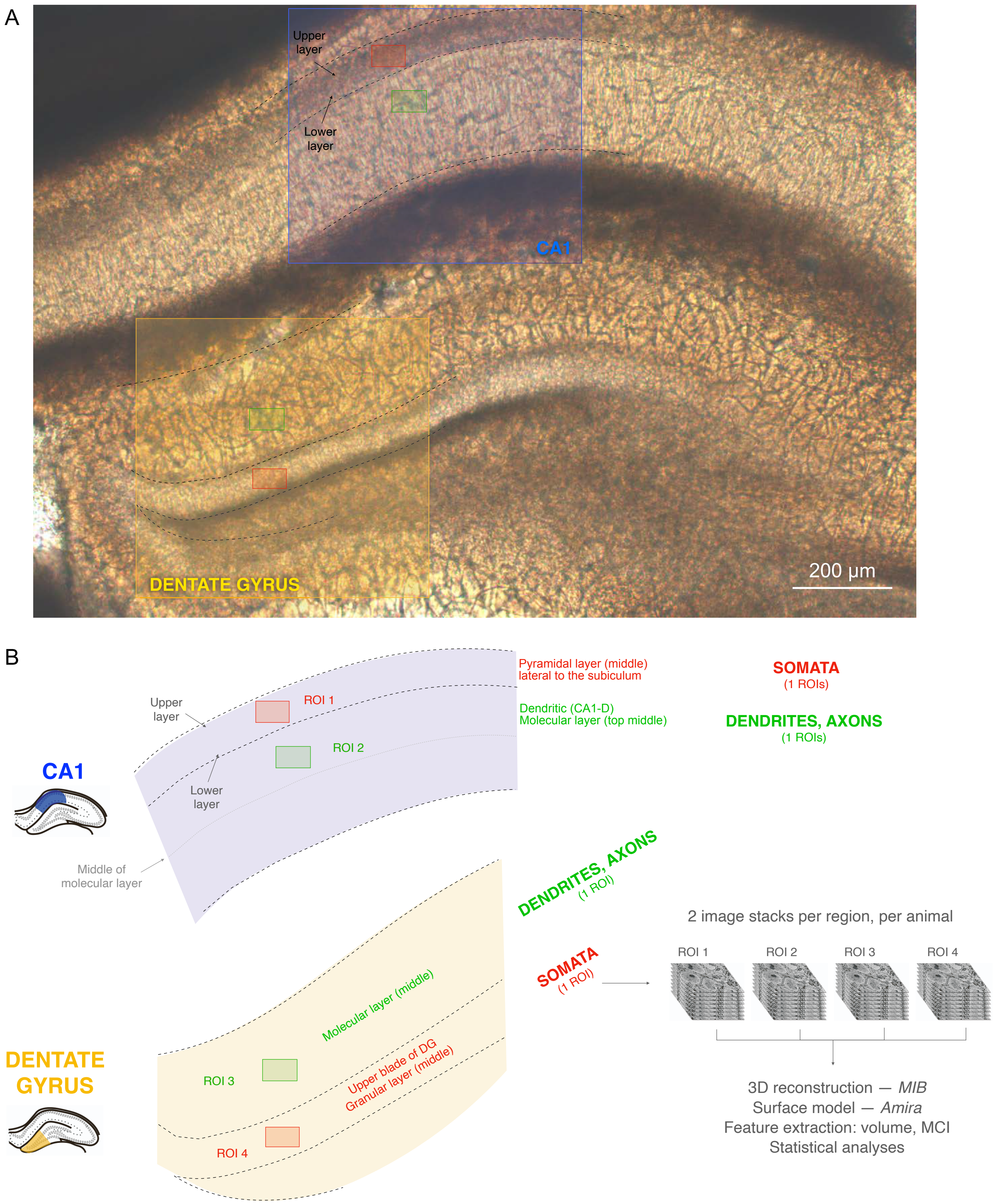

Figure S1. Selection of regions of interest for SBF-SEM of the mouse hippocampus. Related to Figure 1.

(A) Bright field imaging of the fixed dorsal hippocampus. The DG and CA1 regions used in analyses are highlighted, and the regions of interest imaged by SBF-SEM are shown as green (molecular layer) and red (granular and pyramidal layers) (dimensions: 40 µm x 40 µm). Scale bar, 200 µm.

(B) Same regions as in (A) showing anatomical landmarks used for imaging. For each brain region (CA1, DG) and each sub-region (molecular layer, granular/pyramidal layer) of each mouse 2 ROIs were captured, for a total of 4 ROIs per animal. Somata were analysed from ROIs from the granular layer in DG and from the pyramidal cell layer in CA1. Dendrites and axons were analyzed from the middle of the granular layer of CA1 near the subiculum and from the upper blade of the DG. Experiments included 7 mice (3 young, 4 old), for a total 28 ROIs and 3D reconstructions from which statistical analyses were performed.

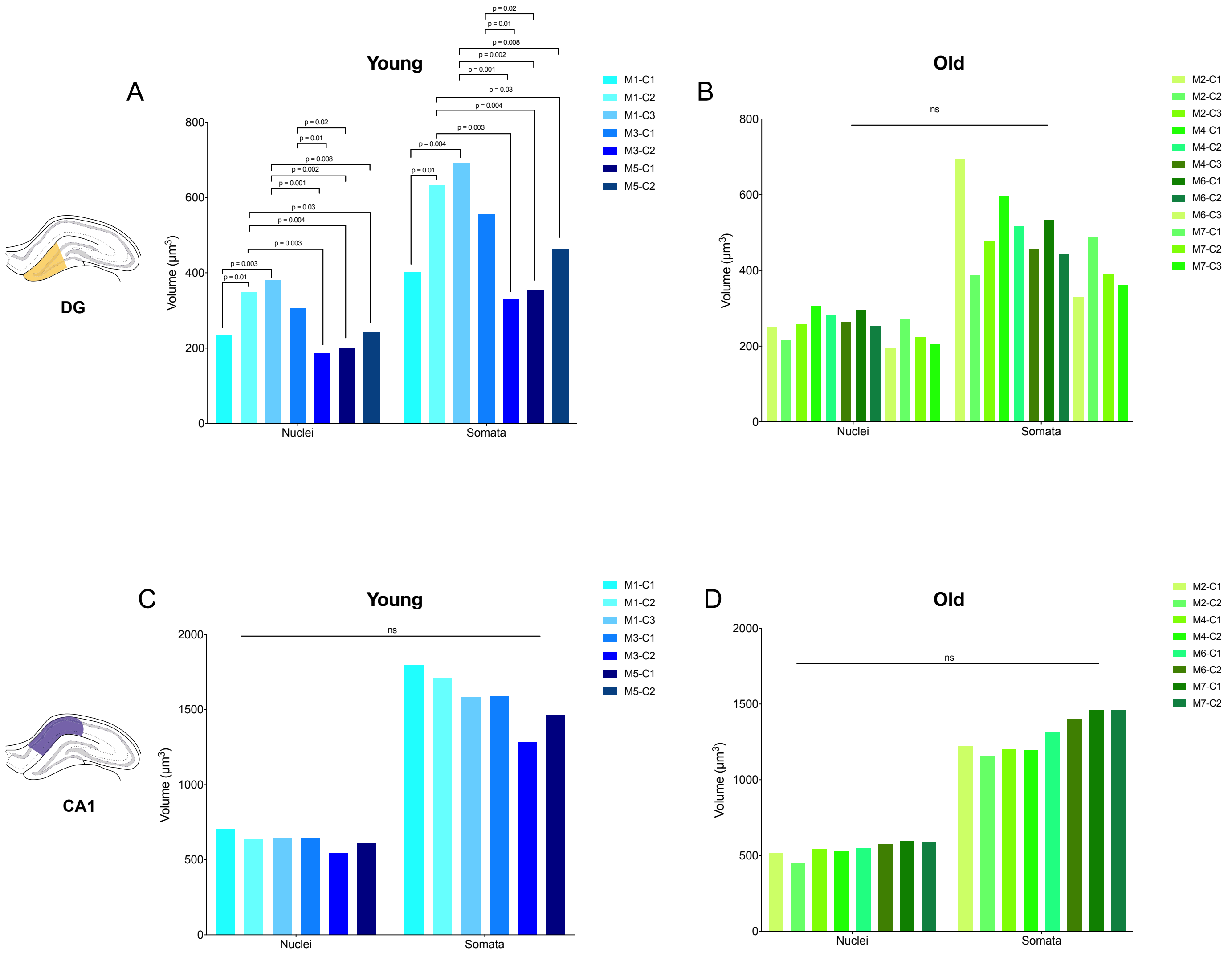

Figure S2. Cell-to-cell differences within the mouse DG and CA1. Related to Figure 1.

(A-B) Average nuclei and somata volume in individual granule cells in young **(A)** and old **(B)** in the DG.

(C-D) Average nuclei and somata volume in individual pyramidal cells in young **(C)** and old **(D)** in the CA1.

DG: n=7 young, n=12 old somata and nuclei; CA1: n=6 young, n=8 old somata and nuclei. Two–Way ANOVA followed by post-hoc tests using the two-stage step up method of Benjamini, Krieger, and Yekutieli to correct for multiple comparisons ($p < 0.05$, $q < 0.05$).

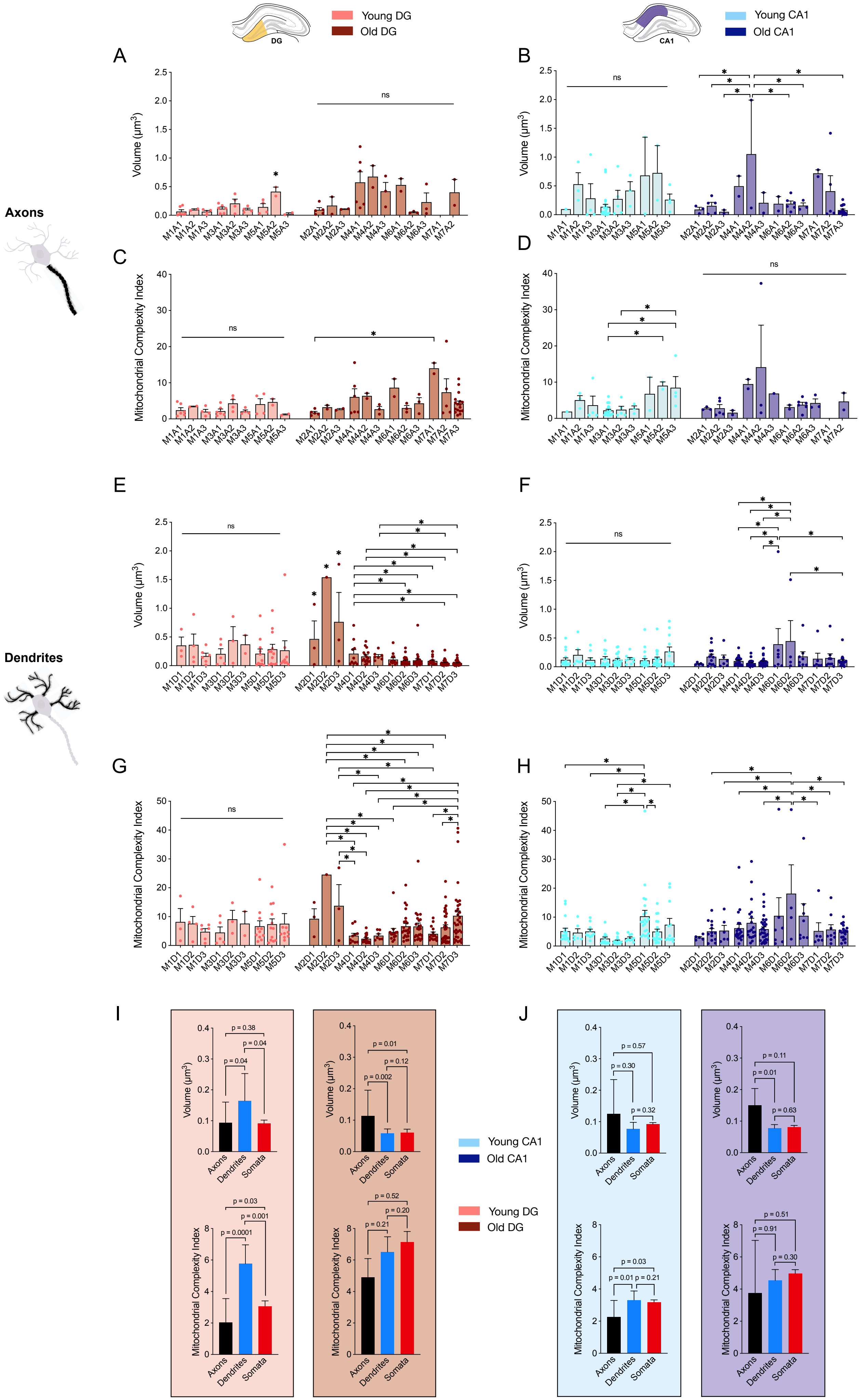

DG
Young DG
Old DG
CA1
Young CA1
Old CA1
A
B
C
D
E
F
G
H
I
J
Axons
Dendrites
Volume (μm³)
Mitochondrial Complexity Index
ns
*
M1A1 M1A2 M1A3 M3A1 M3A2 M3A3 M5A1 M5A2 M5A3
M2A1 M2A2 M2A3 M4A1 M4A2 M4A3 M6A1 M6A2 M6A3 M7A1 M7A2 M7A3
M1D1 M1D2 M1D3 M3D1 M3D2 M3D3 M5D1 M5D2 M5D3
M2D1 M2D2 M2D3 M4D1 M4D2 M4D3 M6D1 M6D2 M6D3 M7D1 M7D2 M7D3
Axons
Dendrites
Somata
p = 0.38
p = 0.04
p = 0.04
p = 0.03
p = 0.001
p = 0.0001
p = 0.01
p = 0.12
p = 0.002
p = 0.52
p = 0.20
p = 0.21
p = 0.57
p = 0.30
p = 0.32
p = 0.03
p = 0.01
p = 0.21
p = 0.11
p = 0.01
p = 0.63
p = 0.51
p = 0.91
p = 0.30

Figure S3. Mitochondrial heterogeneity and effect aging across sub-cellular compartments. Related to Figure 4.

(A-D) Comparison of the volume **(A-B)** and MCI **(D-E)** in each DG and CA1 axons of young and old mice.

(E-H) Comparison of the volume **(E-F)** and MCI **(G-H)** in each DG and CA1 dendrites of young and old mice.

(I) Average mitochondrial volume and MCI in DG of axons, dendrites and somata of young and old animals. Left panel young animals - right panel old animals.

(J) Average mitochondrial volume and MCI in CA1 of axons, dendrites and somata of young and old animals. Left panel young animals - right panel old animals.

Young DG: n= 34 axonal; n=55 dendritic; n=471 somatic mitochondria; old DG: n= 52 axonal; n=184 dendritic; n=777 somatic mitochondria. Young CA1: n= 48 axonal; n=131 dendritic; n=1,588 somatic mitochondria. Old CA1: n= 37 axonal; n=146 dendritic; n=1,930 somatic mitochondria. n=3 axons, dendrites, and somata were analyzed for each region (DG and CA1), in young and old (total 12 of each).

Data are presented as median with 95% C.I. Kruskal-Wallis test followed by post-hoc tests using the two-stage step up method of Benjamini, Krieger, and Yekutieli to correct for multiple comparisons (* $p < 0.05$, $q < 0.05$).

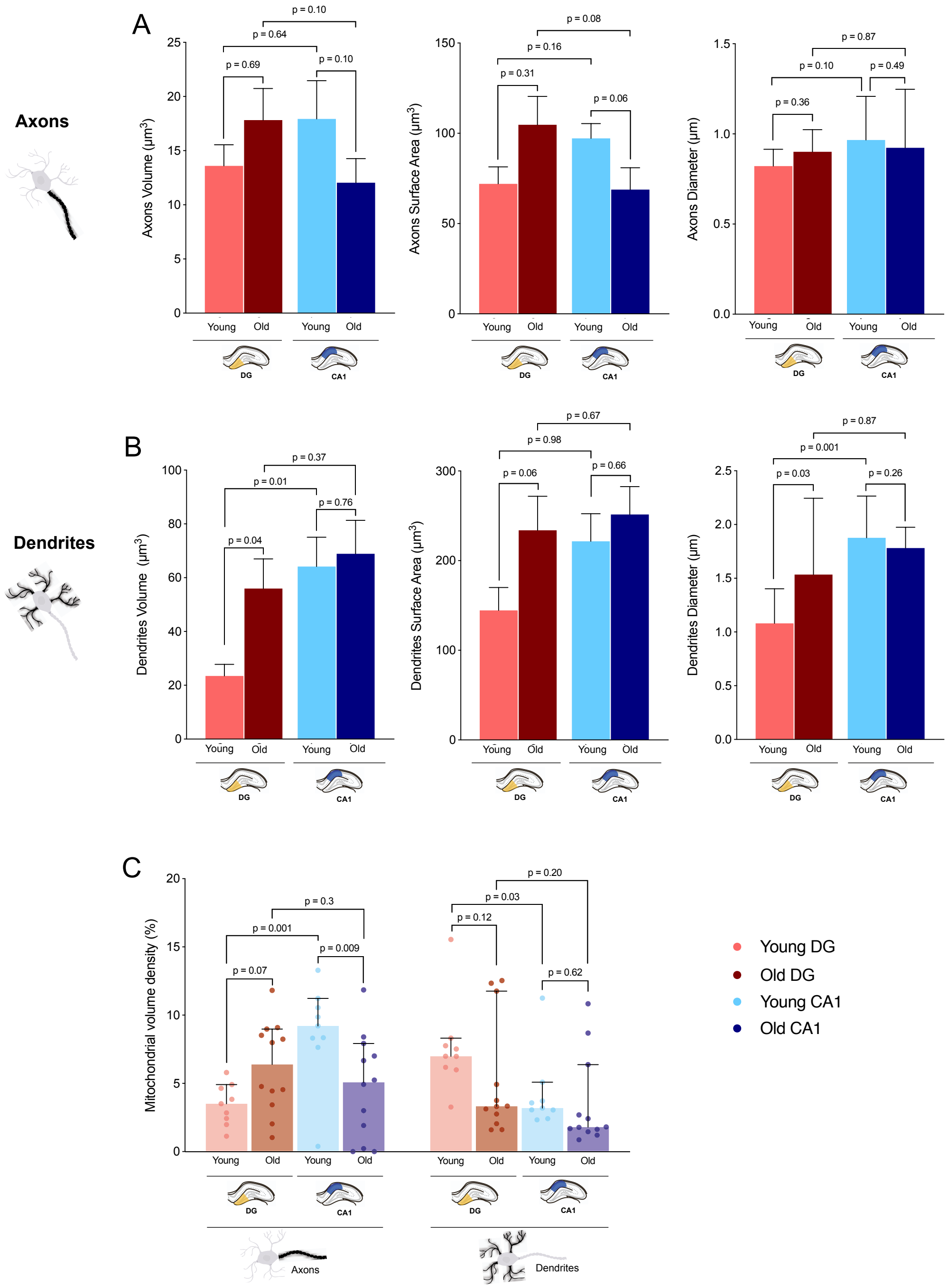

Figure S4. Effect of aging on axons and dendrites volume, surface area, diameter and mitochondrial volume density, within the DG and CA1. Related to Figure 4.

(A-B) Average axon **(A)** and dendrite **(B)** volume, surface area and diameter of young and old animals in DG and CA1.

(C) Mitochondrial volume density (MVD) in axons and dendrites in DG and CA1 for young and old mice.

Young DG: n=9 axons, n=9 dendrites; old DG: n=12 axons; n=12 dendrites; young CA1: n=9 axons; n=9 dendrites; old CA1: n= 10 axons; n=12 dendrites. Data are presented as median with 95% CI. Kruskal-Wallis test followed by post-hoc tests using the two-stage step up method of Benjamini, Krieger, and Yekutieli to correct for multiple comparisons ($p < 0.05$, $q < 0.05$).

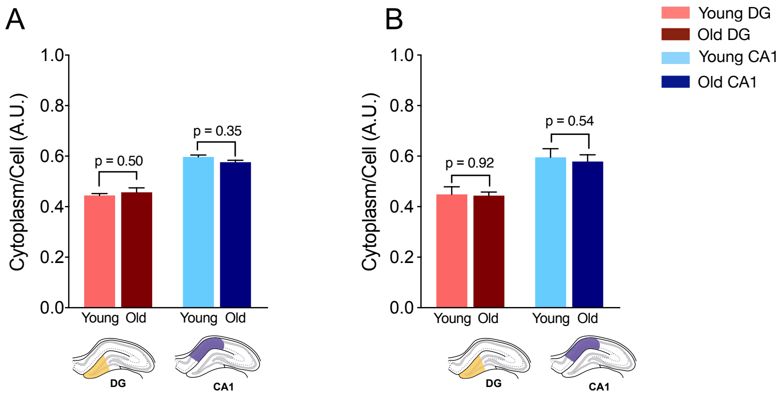

Figure S5. Effect of aging on nuclear/cell volume ratio within DG and CA1 region. Related to Figure 5.

(A) Ratio nuclear/cell and **(B)** cytoplasm/cell volume in young and old animals for the DG and CA1 regions. DG: n=7 young, n=12 old somata; CA1: n=6 young, n=8 old somata. Data are presented as median with 95% CI. Kruskal-Wallis test followed by post-hoc tests using the two-stage step up method of Benjamini, Krieger, and Yekutieli to correct for multiple comparisons ($p < 0.05$, $q < 0.05$).
